# Determinants of breastfeeding discontinuation in an Italian cohort of mother-infant dyads in the first six months of life: a randomized controlled trial

**DOI:** 10.1186/s13052-018-0572-z

**Published:** 2018-11-06

**Authors:** Fabio Mosca, Paola Roggero, Francesca Garbarino, Daniela Morniroli, Beatrice Bracco, Laura Morlacchi, Domenica Mallardi, Maria Lorella Gianni, Dario Consonni

**Affiliations:** 1 NICU Fondazione IRCCS Ca’ Granda Ospedale Maggiore Policlinico, via Commenda 12, Milano, Italy; 2 Department of Clinical Sciences and Community Health, University of Milan, via San Barnaba 8, 20122 Milan, Italy; 3 Fondazione IRCCS Ca’ Granda Ospedale Maggiore Policlinico Epidemiology Unit, via San Barnaba 8, 20122 Milan, Italy

**Keywords:** Breastfeeding, Breastfeeding rates, Term infant, Breast milk substitutes

## Abstract

**Background:**

Among breastfeeding determinants, the marketing of breast milk substitutes might contribute to suboptimal breastfeeding rates. The aim of this study was to investigate the effect of receiving information on breast milk substitutes on breastfeeding rates.

**Methods:**

We conducted a randomized, single-blind, controlled trial from 2012 to 2014 in a northern Italian maternity ward. We enrolled 802 Caucasian mothers who gave birth to healthy, full-term singletons with a birth weight > 2500 g and who were exclusively breastfeeding from delivery to discharge. Mothers who gave birth to infants with congenital diseases, chromosomal abnormalities, perinatal infections and/or cardio-respiratory instability and/or mothers being affected by endocrine and/or metabolic and/or gastrointestinal and/or renal diseases were excluded.

Mothers were randomized to either receive (group A, *n* = 405) or not (group B, *n* = 397) written information on a breast milk substitute at discharge. Breastfeeding was promoted and supported in all mother-infant pairs equally. The mode of feeding for up to 6 months after delivery was determined by phone interview. To detect a 10% difference between groups in the discontinuation rate of exclusive breastfeeding at three months of age at 5% significance and 80% power, a total of 356 mother-infant pairs per group were needed.

**Results:**

The exclusive breastfeeding prevalence was 91% and 92% at 7 days, 79% and 70% at 1 month, 75% and 66% at 2 months, 72% and 62% at 3 months, and 3% and 2% at 6 months in groups A and B, respectively. The relative risk (95% confidence interval) of exclusive breastfeeding (group A vs B) at 7 days and at 1, 2, 3 and 6 months was as follows: 0.99 (0.95–1.03), 1.12 (1.03–1.21), 1.13 (1.03–1.24), 1.15 (1.04–1.27), and 1.49 (0.62–3.61).

Nutritional, lifestyle and lactational factors were the primary contributing determinants to early breastfeeding discontinuation.

**Conclusions:**

The present findings indicate that receiving written information on breast milk substitutes at hospital discharge, provided that breastfeeding support and education are offered, does not negatively affect breastfeeding rates.

**Trial registration:**

NCT03208114. Registered 5 July 2017.

## Background

Evidence indicates that not breastfeeding or early breastfeeding cessation are associated with health risks for both mothers and infants [1, 2]. The World Health Organization recommends exclusive breastfeeding for six months, with breastfeeding continuing to be an important part of the diet until at least two years of age [3]. However, current breastfeeding rates in many countries are far from the recommended targets [2, 4]. In Italy, according to the Italian National Statistics Institute [5], 48.7% of infants are being exclusively breastfed in the first month, with values falling to 43.9% within the first three months and to 5.5% at six months of life. A survey conducted in 2012 in Lombardy [6] reported a progressive reduction of exclusive breastfeeding rates from 67.3% at hospital discharge to 47.3% and 27% within 120 and 180 days, respectively, after delivery.

The determinants of breastfeeding have been extensively investigated in order to refine breastfeeding promotion policies, interventions and programmes [4, 7]. Rollins et al. described a conceptual model in which structural, setting and individual determinants are crucial for creating a supportive breastfeeding environment [4]. Among the recommended interventions to protect, promote and support breastfeeding, baby friendly support enhances exclusive breastfeeding by 49% within the first 5 months and any breastfeeding by 66% up to six months [4].

Greater political commitment has been advocated towards the implementation of the International Code of Marketing of Breastmilk Substitutes, which aims to enable parents to make infant feeding choices without exposure to commercial bias and with a complete understood of what is in their child’s best interest [7–10]. Indeed, breast milk substitute marketing to and through health facilities and health care providers has been reported to contribute to suboptimal breastfeeding rates [11, 12].

To the best of our knowledge, there is a paucity of data regarding the effect of written information on breast milk substitutes written on a new-born’s discharge medical documents on subsequent breastfeeding rates.

The aim of our study was to investigate the effect of information on breast milk substitutes written on a new-born’s discharge documents on breastfeeding rates in a cohort of mothers who were exclusively breastfeeding at hospital discharge. We tested the hypothesis that information on breast milk substitutes would negatively affect breastfeeding rates. The secondary aim of the study was to investigate the main reasons for breastfeeding discontinuation over the first six months after delivery.

## Methods

### Ethics statement

The study was approved by the Ethics Committee of the Fondazione IRCCS Ca′ Granda Ospedale Maggiore Policlinico, and written informed consent was obtained from the parents.

### Subjects

All consecutive mothers that delivered to Fondazione IRCCS Ca′ Granda Ospedale Maggiore from 2012 to 2014, were screened for eligibility. Inclusion criteria were: being of Caucasian race, have given birth to healthy, full-term infants with a birth weight > 2500 g after a singleton pregnancy, exclusively breastfeeding during their hospital stay. Exclusion criteria were: mothers who presented contraindications to breastfeeding or who had chosen not to breastfeed and mothers of newborns admitted to Neonatal Intensive Care Unit and affected by any condition that could interfere with breastfeeding including congenital diseases, chromosomal abnormalities, lung, brain, metabolic, cardiac or gastrointestinal diseases.

### Design

**Fig. 1:**
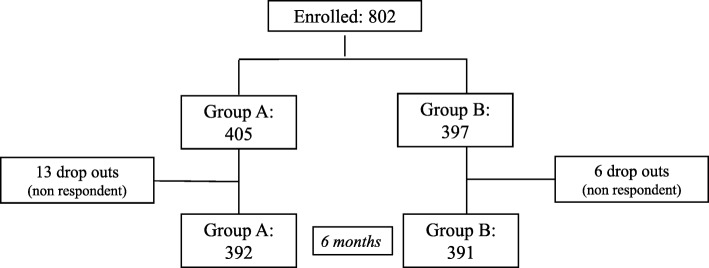
Flow chart of the study

We conducted a controlled, single-blind, randomized trial. Breastfeeding was promoted and supported in all mother-infant pairs throughout the hospital stay following the Ten Steps to Successful Breastfeeding [13]. Enrolment and randomization were performed at discharge. The flow chart of the study is provided in Fig. 1.

### Randomization and masking

Mothers were randomized either to receive written information on the name of the starter formulas commercially available in Italy on the infant’s discharge document (group A) or not to receive this information (group B).

Randomization was performed by an independent investigator with a random permuted block size of 4. Phone interviews were performed by five investigators who were blinded to the randomization.

### Procedures

At enrolment, the following maternal variables were collected through a face-to-face interview: mode of delivery; parity; previous breastfeeding experience; labour duration; drug administration during pregnancy and/or labour, including analgesia or anaesthesia; diseases diagnosed during pregnancy; maternal education classified as low (≤13 years) or high (> 13 years); pre-pregnancy body mass index; weight gain during pregnancy; marital status; age; and attitude towards smoking. Mothers were also asked whether they had attended a pre-pregnancy course, had intended to breastfeed and were satisfied with the breastfeeding support received during their hospital stay. Timing of the first latch was also registered.

The following neonatal variables were collected: gestational age, gender, Apgar scores at 1 and 5 min, birth weight, length and head circumference.

Mothers were contacted by phone call at 7 (±3) days and at 1 (±7 days), 2 (±7 days), 3 (±7 days), and 6 (±7 days) months after delivery [14, 15]. Following a structured interview, mothers were asked whether their infant had been breastfed during the last 24 h and had been fed any water, fruit juice, formula, semi-solid foods and/or solid foods during the last 24 h. If mothers reported that complementary feeding had been started, they were then asked when it was first introduced.

Mode of feeding was categorized according to the World Health Organization [16].

If the infant was no longer breastfed, the mothers were administered a questionnaire modified after that reported by Odom et al. [17] to investigate the main reasons for breastfeeding discontinuation. The questionnaire focused on 7 macro areas (Table 1).

**Table 1:** Questionnaire investigating the main reasons associated with breastfeeding discontinuation

Lactational factors	
Infant’s difficulty in sucking or latching	
Presence of nipple cracks and fissures	
Presence of breast engorgement or mastitis	
Painful breastfeeding	
Delayed lactogenesis II	
Milk pumping factors	
Mother could not or intended not to pump or breastfeed at work	
Pumping was too tiring and time consuming	
Psychosocial factors	
Breastfeeding was too inconvenient	
Breastfeeding was too tiring	
Need or wish to have the infant fed by someone else	
Nutritional factors	
According to my opinion, breast milk did not satisfy the infant’s nutritional requirements	
Perception of inadequate milk supply	
A health professional said the baby was not gaining enough weight	
Lifestyle factors	
Wish to lose weight	
Wish to stop breastfeeding	
Wish to follow the usual diet	
Wish to smoke again or more than allowed while breastfeeding	
Medical factors	
New pregnancy or wish to have a new pregnancy	
Drug consumption and/or maternal disease	
The infant was sick and could not be breastfed	
Factors related to the infant	
The baby began to bite	
The baby lost interest in nursing or began to wean him or herself	
The infant was growing, and the difference between breast milk and formula was no longer important	

Mothers were asked to rate the importance of each item in the questionnaire according to a 5-point Likert scale: 1 = not at all important, 2 = not very important, 3 = moderately important, 4 = important, and 5 = extremely important.

### Adverse events

Adverse events were assessed based on inquiries to the mothers and evaluated by the investigator for severity. An adverse event was defined as any event that was not consistent with the information provided in the consent form or that could not reasonably be expected to accompany the natural history and progression of the subject’s condition throughout the study. Adverse events were considered serious if they resulted in death or were life-threatening, required hospitalization or surgical intervention, resulted in persistent or significant disability/incapacity or, based on medical judgment, could jeopardize the patient and require medical or surgical intervention to prevent one of the outcomes listed above. All other adverse events were categorized as non-serious.

### Statistical analysis

#### Sample size

Assuming a 70% exclusive breastfeeding rate at three months in mother-infant pairs not receiving information on breast milk substitutes at discharge, a total of 356 mother-infant pairs per group were needed to detect a 10% difference between groups in the discontinuation rate of exclusive breastfeeding at three months of age at 5% significance and 80% power.

Descriptive data are presented as the mean ± SD or number (percentage) of observations. With regard to items scored on a 5-point Likert scale, the answers were categorized as not important (scores 1 and 2) or important (scores 3, 4 and 5) for the analysis.

For comparisons between the two groups, we used Student’s t test and the chi-squared test for quantitative and categorical variables, respectively. We also calculated the relative risk (RR) and 95% confidence interval (CI) of exclusive breastfeeding (group A vs B) at the various follow-up times. All statistical analyses were performed with SPSS (version 12, SPSS, Chicago, IL).

## Results

A total of 802 mother-infant pairs were enrolled (Fig. 1). The basic characteristics according to group are shown in Table 2. No difference between groups was found in the basic characteristics of the mother-infant pairs, except for the rate of caesarean section, which was higher in group B than in group A.

**Table 2:** Basic characteristics of the enrolled mother-infant dyads according to group

Mothers			
Mean ± SD	Group A (*n* = 405)	Group B (*n* = 397)	P
Age (years)	35.1 ± 4.8	34.8 ± 4.6	0.4
Pre-pregnancy maternal body mass index (kg/m^2^)	21.5 ± 3.3	21.7 ± 3.5	0.3
Weight gain during pregnancy (kg)	12.4 ± 4.8	13.0 ± 6.9	0.1
Duration of previous breastfeeding experience (months)	8.3 ± 6.7	7.2 ± 6.0	0.1
Labour duration (hours)	5.4 ± 5.8	5.1 ± 5.9	0.5
Timing of first latch (minutes)	120.4 ± 286	113.6 ± 193	0.6
N (%)			
Marital status			0.8
Married	283 (70)	281 (71)	
Not married	121 (30)	114 (29)	
Maternal education			0.6
Low maternal education (≤13 years)	25 (6)	21 (6)	
High maternal education (> 13 years)	379 (94)	374 (94)	
Parity			0.4
Primiparous	235 (58)	245 (62)	
Multiparous	169 (42)	152 (38)	
Attitude towards smoking	21 (5)	14 (3.5)	0.2
Prenatal intention to breastfeed	405 (100)	394 (99)	0.3
Previous breastfeeding experience	138 (34)	118 (30)	0.6
Attendance at a pregnancy course	174 (43)	183 (46)	0.3
Diseases during pregnancy	14 (3)	16 (4)	0.7
Drug consumption during pregnancy	128 (32)	128 (32.2)	0.8
Anaesthesia/analgesia during labour	320 (79)	334 (84)	0.06
Mode of delivery			0.003
Caesarean section	147 (36)	185 (47)	
Vaginal	258 (64)	212 (53)	
Satisfaction with breastfeeding support received during hospital stay	379 (94)	360 (91)	0.2
Infants			
Mean ± SD	Group A (*n* = 405)	Group B (*n* = 397)	P
Gestational age (weeks)	38.9 ± 1.05	38.9 ± 1.1	0.7
1-min Apgar score	9.01 ± 0.70	8.9 ± 0.74	0.3
5-min Apgar score	9.9 ± 0.34	9.8 ± 0.36	0.4
Birth weight (g)	3312 ± 366	3332 ± 330	0.4
Birth length (cm)	49.7 ± 1.6	49.8 ± 1.8	0.6
Head circumference (cm)	34.5 ± 1.3	34.6 ± 1.6	0.3
N (%)			
Gender			0.3
Male	209 (52)	192 (48)	
Female	195 (48)	205 (52)	

Breastfeeding rates according to group are shown in Table 3. The rate of exclusive breastfeeding was significantly higher in group A than in group B at 1, 2 and 3 months after delivery. At 6 months, exclusive breastfeeding was negligible in both groups. The relative risk (95% CI) of exclusive breastfeeding (group A vs B) at 7 days and at 1, 2, 3 and 6 months was as follows: 0.99 (0.95–1.03), 1.12 (1.03–1.21), 1.13 (1.03–1.24), 1.15 (1.04–1.27), and 1.49 (0.62–3.61).

**Table 3:** Breastfeeding rates according to group and mode of delivery

	All mother-infant dyads			Vaginal delivery			Caesarean section		
	Group A	Group B	P	Group A	Group B	P	Group A	Group B	P
7 days	*N* = 405 (100%)	*N* = 397 (100%)		*N* = 258 (100%)	*N* = 212 (100%)		*N* = 147 (100%)	*N* = 185 (100%)	
Exclusive breastfeeding	368 (90.9)	364 (91.7)	0.7	242 (93.8)	194 (91.5)	0.34	126 (85.7)	170 (91.8)	0.07
Predominant breastfeeding	0	0		0	0		0	0	
Mixed breastfeeding	32 (7.9)	28 (7.0)		14 (5.4)	15 (7.1)		18 (12.2)	13 (7.1)	
Exclusive formula	5 (1.2)	5 (1.3)		2 (0.8)	3 (1.4)		3 (2.1)	2 (1.1)	
1 month	*N* = 396	*N* = 396		*N* = 254	*N* = 212		*N* = 142	*N* = 184	
Exclusive breastfeeding	311 (78.6)	278 (70.2)	0.007	206 (81.1)	149 (70.3)	0.006	105 (73.9)	129 (70.2)	0.44
Predominant breastfeeding	0	0		0	0		0	0	
Mixed breastfeeding	56 (14.1)	77 (19.4)		32 (12.6)	44 (20.7)		24 (16.9)	33 (17.9)	
Exclusive formula	29 (7.3)	41 (10.4)		16 (6.3)	19 (9.0)		13 (9.2)	22 (11.9)	
2 months	*N* = 392	*N* = 391		*N* = 251	*N* = 209		*N* = 141	*N* = 182	
Exclusive breastfeeding	294 (75)	259 (66.2)	0.007	196 (78.1)	138 (66.1)	0.004	98 (69.6)	121 (66.5)	0.56
Predominant breastfeeding	3 (0.8)	9 (2.3)		2 (0.8)	7 (3.3)		1 (0.8)	2 (1.1)	
Mixed breastfeeding	47 (12)	58 (14.8)		26 (10.3)	33 (15.8)		21 (14.8)	25 (13.7)	
Exclusive formula	48 (12.2)	65 (16.7)		27 (10.8)	31 (14.8)		21 (14.8)	34 (18.7)	
3 months	*N* = 392	*N* = 391		*N* = 251	*N* = 209		*N* = 141	*N* = 182	
Exclusive breastfeeding	281 (71.7)	244 (62.4)	0.006	186 (74)	134 (64)	0.02	95 (67.4)	110 (60.5)	0.19
Predominant breastfeeding	8 (2)	12 (3)		7 (3)	7 (3)		1 (0.7)	5 (2.7)	
Mixed breastfeeding	38 (9.7)	52 (13.3)		20 (8)	27 (13)		18 (12.8)	25 (13.7)	
Exclusive formula	64 (16.3)	82 (21)		38 (15)	40 (19)		26 (18.4)	42 (23.1)	
Weaning during breastfeeding	0	0		0	0		0	0	
Weaning during formula feeding	1 (0.3)	0		0	0		1 (0.7)	0	
Weaning during mixed breastfeeding	0	1 (0.3)		0	1 (0.48)		0	0	
6 months	*N* = 391	*N* = 389		*N* = 250	*N* = 207		*N* = 141	*N* = 182	
Exclusive breastfeeding	12 (3.1)	8 (2)	0.37	5 (2.0)	2 (0.9)	0.4	7 (5.0)	6 (3.3)	0.44
Predominant breastfeeding	1 (0.3)	1 (0.3)		1(0.4)	1 (0.5)		0	0	
Mixed breastfeeding	1 (0.3)	0		0	0		1 (0.7)	0	
Exclusive formula	4 (1)	9 (2.3)		3 (1.0)	4 (1.9)		1 (0.7)	5 (2.7)	
Weaning during breastfeeding	228 (58.3)	201 (51.7)		152 (60.8)	108 (52.2)		76 (53.9)	93 (51.1)	
Weaning during formula feeding	112 (28.6)	133 (34.2)		70 (28.0)	71 (34.3)		42 (29.8)	62 (34.1)	
Weaning during mixed breastfeeding	33 (8.4)	37 (9.5)		19 (7.6)	21 (10.2)		14 (9.9)	16 (8.8)	

Data are presented as the number of observations (%)

Weaning began in groups A and B at 168 ± 14.5 and 168.02 ± 15.8 days (*p* = 0.69), respectively.

Since groups A and B differed with regard to the mode of delivery, the breastfeeding rates were further analysed after stratifying the mothers according to the mode of delivery. Rates of exclusive breastfeeding according to group and mode of delivery are shown in Table 3. With regards to mothers that delivered vaginally, the rate of exclusive breastfeeding was higher at 1, 2 and 3 months in group A than in group B but was similar between groups at 6 months. The relative risk (95% CI) of exclusive breastfeeding (group A vs B) at 7 days and at 1, 2, 3 and 6 months among mothers that delivered vaginally was as follows: 1.03 (0.97–1.08), 1.15 (1.04–1.28), 1.18 (1.05–1.33), 1.16 (1.02–1.31), and 2.07 (0.40–10.6). With regard to mothers that delivered via caesarean section, there was no difference in the breastfeeding rate between groups. The relative risk (95% CI) of exclusive breastfeeding (group A vs B) at 7 days and at 1, 2, 3 and 6 months among mothers that delivered via caesarean section was as follows: 0.93 (0.86–1.01), 1.05 (0.92–1.21), 1.05 (0.90–1.21), 1.11 (0.95–1.31), and 1.51 (0.52–4.38).

The reasons reported by mothers as important in determining early discontinuation of breastfeeding are reported in Table 4. Nutritional factors (“According to my opinion”, “breast milk did not satisfy the infant’s nutritional requirements”, “Perception of inadequate milk supply”, “A health professional said the baby was not gaining enough weight”) were among the most important contributing factors at each study point, and the percentage of mothers that rated them as important ranged from 36 to 99%. Among the lifestyle factors, the item “Wish to not breastfeed” was reported as important in 57% to 37% of cases at three and six months, respectively.

**Table 4:** Reasons stated by the mothers as important in determining early discontinuation and complementation of exclusive breastfeeding

Study time point	7 days	1 month	2 months	3 months	6 months
Lactational factors	%	%	%	%	%
Infant’s difficulty in sucking or latching	10	17	2	22	8
Presence of nipple cracks and fissures	10	10	19	13	6
Presence of breast engorgement or mastitis	10	10	10	14	7
Painful breastfeeding	10	8	8	10	6
Delayed lactogenesis II	22	16	14	19	10
Milk pumping factors					
Mother could not or intended not to pump or breastfeed at work	/	1	2	3	1
Pumping was too tiring and time consuming	10	6	1	9	5
Psychosocial factors					
Breastfeeding was too inconvenient	/	1	1	1	1
Breastfeeding was too tiring	10	13	5	18	10
Too many household duties	/	3	3	4	9
Need or wish to have the infant fed by someone else	10	15	17	29	22
Wish not to breastfeed in public	/	1	1	1	2
Nutritional factors					
According to my opinion, breast milk did not satisfy the infant’s nutritional requirements	49	43	42	62	38
Perception of inadequate milk supply	40	68	67	99	55
A health professional said the baby was not gaining enough weight	40	36	36	51	29
Lifestyle factors					
Wish to lose weight	/	1	1	1	1
Wish to stop breastfeeding	49	40	38	57	37
Wish to follow the usual diet	0	0	0	0	0
Wish to smoke again or more than allowed while breastfeeding	0	0	0	0	0
Medical factors					
New pregnancy or wish to have a new pregnancy	/	1	2	3	4
Drug consumption and/or maternal disease	/	1	1	1	1
The infant was sick and could not be breastfed	/	3	2	5	2
Factors related to the infant					
The baby began to bite	/	6	6	11	6
The baby lost interest in nursing or began to wean him or herself	/	1	1	2	3
The infant was growing. and the difference between breast milk and formula was no longer important	10	5	5	8	8

Lactational factors also scored as important by 5% to 22% of mothers. Milk pumping factors were rated as important by only a limited percentage of mothers. Among the psychosocial factors, the items “Breastfeeding was too tiring” and “Need or wish to have the infant fed by someone else” were the most frequently reported. Items related to the inconvenience of breastfeeding and the wish to not breastfeed in public were reported as important in very few cases. Both medical factors and factors related to the infant were less frequently reported. The item “The infant was growing, and the difference between breast milk and formula was no longer important” was indicated as important by 8% of mothers at three and six months.

### Adverse events

In total, 128 adverse events occurred in 112 infants. Of these, 32 were categorized as serious. The documented reasons for the adverse events were mostly illnesses that are common during the first six months of life (i.e., lower and upper respiratory tract infections, gastroenteritis, and urinary tract infection). There was no difference between the two study groups in the occurrence of adverse events during the study.

## Discussion

These findings indicate that, once adequate breastfeeding support during hospital stay is offered, written information on the name of a breast milk substitute at hospital discharge does not negatively affect exclusive breastfeeding rates in a cohort of mothers who delivered a singleton, full-term infant and who were exclusively breastfeeding during their hospital stay. Contrary to what was expected, among mothers who delivered vaginally, breastfeeding rates were higher at one, two and three months for those who received written information on the name of a breast milk substitute at discharge compared to those that had not received such information. These results could be partially explained by a reduction in maternal anxiety. Mothers, despite receiving breastfeeding counselling during their hospital stay, may be concerned by not receiving instructions on which breast milk substitute they should use in case they perceive having an insufficient milk supply for their infant. Due to the decreased duration of postnatal hospitalization, mothers are discharged from the maternity ward before breastfeeding is fully established, which occurs at three or more days postpartum [18, 19]. Accordingly, the maternal perception that breast milk did not satisfy the infant’s nutritional requirements was one of the main reasons stated by the mothers for early breastfeeding cessation. In line with these findings, Flaherman et al. reported that limited formula supplementation of infants experiencing postnatal weight loss ≥5% during the hospital stay was associated with an increase in breastfeeding rates at three months [20]. The authors hypothesized that the reduced weight loss and signs of infant hunger achieved by formula supplementation decreased maternal concern regarding the adequateness of their milk supply. Maternal anxiety is known to negatively affect lactogenesis [21]. In the present study, contrary to mothers who delivered vaginally, the breastfeeding rates of mothers who delivered by caesarean section were similar among groups at each study point, indicating that caesarean section itself represents a major independent factor that modulates breastfeeding success [22]. However, it must be considered that caesarean section does not appear to negatively influence breastfeeding outcomes at six months once adequate breastfeeding support is provided [23].

Breast milk substitute marketing has been reported to interfere with breastfeeding success. Piwoz et al. performed a review aimed to investigate the extent to which the marketing of breast milk substitutes negatively affects breastfeeding behaviour [11]. The authors reported a negative effect of the promotion of breast milk substitutes by health care professionals on exclusive breastfeeding initiation and duration. In Pakistan, they found that 40% of the mothers were advised by health care professionals during the first six months to feed their infant with formula [24]. Likewise, in Nepal, health care providers recommended formula feeding to 36% of the recently delivered mothers [25]. Sobel et al. [12] reported that, in the Philippines, mothers who received a medical prescription for formula were more likely (odds ratio = 3.25; 95% CI: 1.78–5.91) to use formula, even after adjusting for education and economic factors. The reason for these findings is that health care professionals are regarded as a credible source of information. Furthermore, breast milk substitute marketing can be a significant factor, especially when adequate breastfeeding policies and support are not implemented [11]. However, although information on breast milk substitutes can be regarded as an implicit endorsement of formula feeding, the effect on breastfeeding rates of merely providing information on breast milk substitutes at discharge, given a supportive breastfeeding environment during the hospital stay, has not been previously investigated.

Remarkably, irrespective of having received information about the name of a breast milk substitute, the exclusive breastfeeding rate at six months of age in the present study was far below the recommended target [3]. This result, although in line with the data reported by the Italian National Statistics Institute [5] could be partially explained by the fact that weaning began earlier than recommended in more than half of the enrolled mothers [3, 26]. Contrary to our findings, Cattaneo et al. investigated the efficacy of implementing the Baby Friendly Community Initiative on the exclusive breastfeeding rate at 6 months in 18 Italian Local Health Authorities and reported higher exclusive breastfeeding rates at six months. An early intervention was first performed in the group of Local Health Authorities that had already employed some Baby Friendly Community Initiative activities, followed, after about 1 year, by a late intervention in the remaining ones. Data were collected in all the enrolled Local Health Authorities in three rounds, that is at baseline, after the early intervention period and after the late intervention one. Exclusive breastfeeding rates at the first, second and third round in the Local Health Authorities that underwent the early period of intervention were 58.1%, 57.5% and 62.3% at three months and 9.0%, 7.7% and 7.6% at six months, respectively. Exclusive breastfeeding rates at the first, second and third round in the Local Health Authorities that underwent the late period of intervention were 52.8%, 53.6% and 57.9% at three months and 7.1%, 8.4% and 9.6% at six months, respectively.

The higher exclusive breastfeeding rates at six months may be explained by the particularly strong support for breastfeeding at this study’s setting. However, in the present study, the exclusive breastfeeding rates at 1, 2 and 3 months in both groups were higher than those reported by the Italian National Statistics Institute [5] and by Cattaneo et al. [15]. These findings could reflect the fact that, in the present study, the enrolled mothers did not face breastfeeding difficulties due to having delivered a premature, ill and/or low birth weight baby [27, 28]. Moreover, at six months, breastfeeding rates in infants that had already received complementary foods resulted to be relatively high, being 58% in the mother-infant dyads that had received the information on breast milk substitutes and 52% in the mother-infant dyads that had not received it. These data indicate that breastfeeding was further supported also at community level. Accordingly, the Local Health Authority of Milan has adopted the seven steps of the Baby Friendly Community Initiative [15].

With regard to the reasons stated by the mothers for the early discontinuation of breastfeeding, nutritional factors were among the most contributing factors throughout the study. A mother’s opinion of having an inadequate milk to satisfy her infant’s needs and a perception of an insufficient milk supply were the most frequently cited reasons, whereas the opinion of a health care worker was reported as important in 29% to 51% of cases. Lactational factors also scored as important, particularly during the first three months. These results are consistent with those reported by Odom et al. [17], who investigated the factors associated with not meeting the maternally desired breastfeeding duration. The authors found that breastfeeding duration was shorter than desired for 60% of the enrolled mothers and that the main reasons associated with early cessation were maternal concerns regarding infant nutrition and weight and factors related to lactational problems. Colombo et al. [29] also found that perception of low milk supply, the occurrence of mastitis and nipple fissures represented risk factors for early cessation of breastfeeding. These findings reflect the importance of continued maternal support after hospital discharge by implementing interventions at the community level.

Contrary to what was reported by Odom et al. [17], drug consumption or illness and factors related to milk pumping were reported as important in few cases in the present study probably because we enrolled only healthy full-term infants whereas in the study by Odom et al. [17], infants who completed at least 35 weeks of gestation were included, and some of these infants could have presented with a more immature sucking pattern.

Consistent with the paper by Odom et al. [17], the items “Breastfeeding was too tiring” and “Need or wish to have the infant fed by someone else” were the most frequent psychosocial factors cited as important. Conversely, items related to the inconvenience of breastfeeding and the wish to not breastfeed in public were reported as important in few cases, which could reflect a more positive breastfeeding attitude and culture in our society.

Remarkably, with regard to lifestyle factors, the mothers reported “Wish to stop breastfeeding” as an important determinant in the early cessation of breastfeeding in a high percentage of cases, ranging from 37 to 57%. Among the factors related to the infant, the item “The infant was growing, and the difference between breast milk and formula was no longer important” was the most cited, albeit in a relatively low percentage of cases. These two latter results could stem from the lack of awareness of the dose-dependent manner in which breastfeeding duration determines health benefits, although mothers are educated about the health benefits related to breastfeeding. Efforts should therefore be focused on implementing breastfeeding education interventions.

The strength of the present study is that it enrolled a relatively large sample of mother-infant pairs that were longitudinally followed up for six months. The study present several limitations. First, randomization failed to eliminate confounding factors. However, even though randomization eliminates systematic variation in groups of enrolled subjects, differences between groups may still occur by chance [30]. Second, due to the nature of the trial, it was not possible to perform double-blind randomization. Further, no data related to the socio-economic status was collected although it was expected that randomization would have adjusted for the most important confounding factors. Moreover, no relevant data at four and five months were available even though mothers at the prearranged scheduled phone interview at six months were asked information on mode of feeding during the previous 24 h and, if already introduced, on the timing of complementary feeding initiation. No information on visits to health care providers during the study period are provided. However, it has to be taken into account that the primary aim of the study was not to investigate the role of health care professionals in promoting and supporting breastfeeding after hospital discharge. Moreover, in Italy, according to the National Health System, family paediatricians provide primary care of all patients from birth to 16 years of age, including breastfeeding promotion and support of breastfeeding. Furthermore data refers to a period ranging from 2012 to 2014 so that changes in the procedures adopted by our institution over the last few years could have led to different results. Lastly, this study was a single centre study; therefore, the findings could not be generalized to the general population. Nevertheless, because this study is a single-centre study, the findings were not influenced by inconsistent approaches to breastfeeding promotion and support during hospital stay.

## Conclusions

Contrary to our hypothesis, the present findings indicate that providing written information on breast milk substitutes at hospital discharge did not negatively affect breastfeeding rates during the first six months in a cohort of mothers who delivered a full-term, healthy singleton infant. Health care professionals should strive to protect, promote and support breastfeeding during the hospital stay and at the community level, focusing on the modifiable determinants of suboptimal breastfeeding behaviour.

## Abbreviations

CI: Confidence of interval
RR: Relative risk
